# Use of savolitinib as neoadjuvant therapy for non–small cell lung cancer patient with MET exon 14 skipping alterations: A case report

**DOI:** 10.3389/fonc.2022.968030

**Published:** 2022-09-08

**Authors:** Yu Zhang, Hao Zhang, Hanqing Wang, Jingtong Zeng, Bo Zhang, Ning Zhou, Lingling Zu, Zuoqing Song, Changli Wang, Song Xu

**Affiliations:** ^1^ Department of Lung Cancer, Tianjin Lung Cancer Center, Tianjin Medical University Cancer Institute and Hospital, National Clinical Research Center for Cancer, Key Laboratory of Cancer Prevention and Therapy, Tianjin’s Clinical Research Center for Cancer, Tianjin, China; ^2^ Department of Lung Cancer Surgery, Tianjin Medical University General Hospital, Tianjin, China; ^3^ Tianjin Key Laboratory of Lung Cancer Metastasis and Tumor Microenvironment, Lung Cancer Institute, Tianjin Medical University General Hospital, Tianjin, China

**Keywords:** savolitinib, neoadjuvant, NSCLC, MET exon 14 skipping, targeted therapy

## Abstract

Savolitinib is a tyrosine kinase inhibitor being developed for the treatment of metastatic non–small cell lung cancer (NSCLC) with mesenchymal–epithelial transition (MET) factor exon 14 skipping alterations. However, the role of savolitinib in neoadjuvant therapy for lung cancer remains unclear. Here, we present a case of a 65-year-old woman diagnosed with stage IIIA (cT2bN2M0, eighth TNM stage) upper right lung adenocarcinoma harboring MET exon 14 skipping alterations. After 4 weeks of therapy, a partial response was achieved with neoadjuvant savolitinib, and significant shrinkage in tumor and lymph nodes was observed. We also measured the immune microenvironment of the primary tumor pre- and posttreatment with savolitinib.

## Introduction

Mesenchymal–epithelial transition (MET) exon 14 (METex14) skipping mutations occur in approximately 3% of lung adenocarcinoma patients and 1%–2% of patients with other lung cancer subtypes (1). This gene encodes a member of the receptor tyrosine kinase family of proteins which is the product of the proto-oncogene MET. In 2003 and 2005, Ma et al. reported a series of novel METex14 splicing variants (2, 3). In 2015, Paik et al. demonstrated that mutations of RNA splice acceptor and donor sites involving exon 14 of MET could lead to exon skipping, and the tumors with this mutation could respond to MET-targeted therapies (4).

Savolitinib, approved in June 2021 in China, was used for the treatment of NSCLC with METex14 skipping alterations in patients who are intolerant or whose disease had progressed after platinum-based chemotherapy (5). However, there are no reports regarding neoadjuvant treatment using savolitinib for NSCLC patients with METex14.

## Case report

A 62-year-old female patient with no history of smoking presented with a non-productive cough and bloody sputum for 4 months. Enhanced computed tomography (CT) scan revealed a mass of 43-mm diameter in the right upper lung and enlarged mediastinal lymph nodes (stations 2, 3, and 4). Bronchoscopic biopsy confirmed the diagnosis of adenocarcinoma of clinical stage IIIA (cT2bN2M0) with a Ki-67 score of 40%. Further testing using a real-time PCR (including ALK, ROS1, RET, KRAS, BRAF, NRAS, HER2, PIK3C, MET, and EGFR) detected only METex14 skipping mutation. Multidisciplinary team (MDT) recommended neoadjuvant therapy followed by surgical resection. Savolitinib at a dosage of 250 mg twice daily was prescribed after obtaining informed consent from the patient.

**Figure 1:**
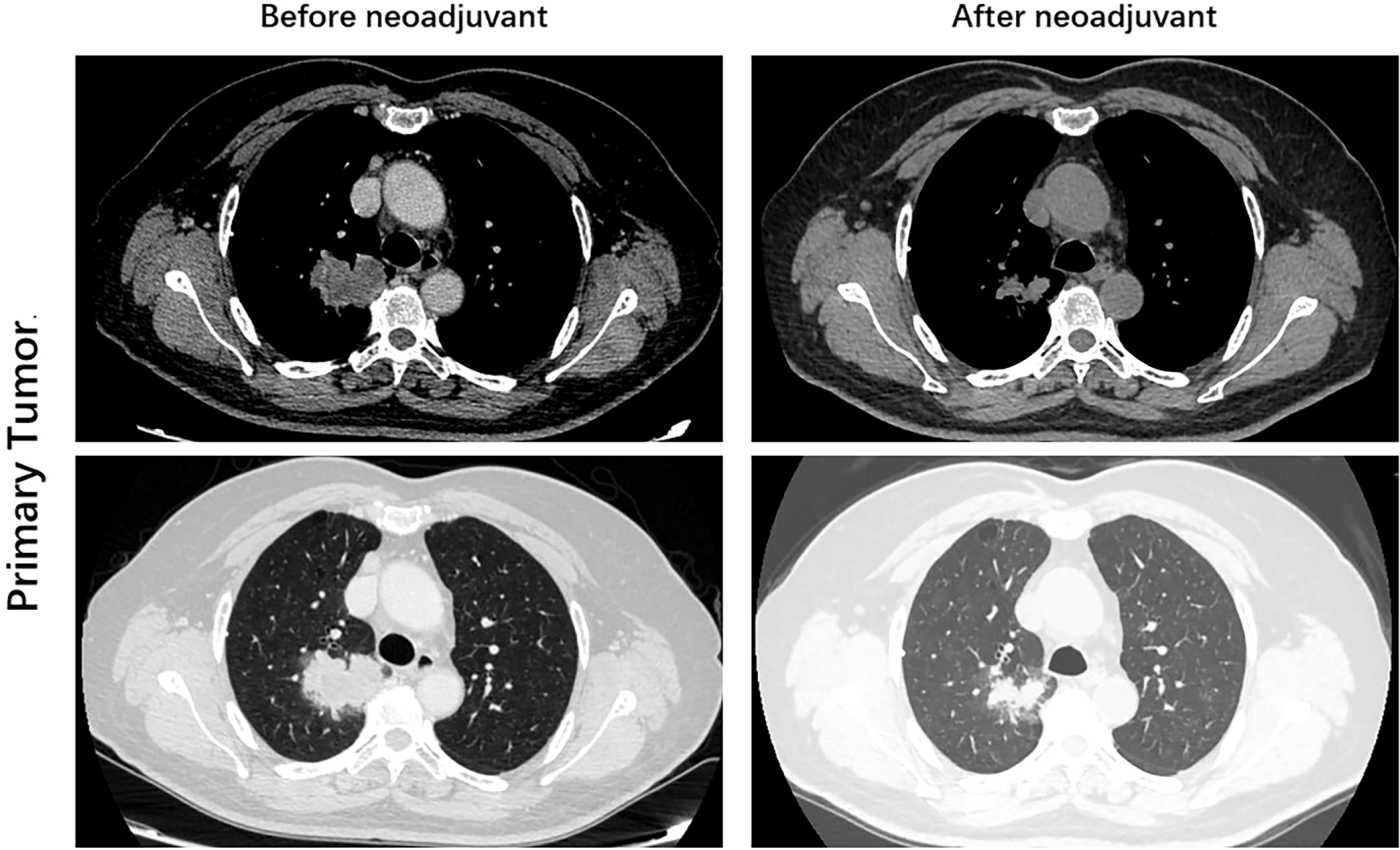
Images during neoadjuvant savolitinib treatment. Enhanced chest CT images of right lung adenocarcinoma before and after the neoadjuvant therapy.

**Figure 2:**
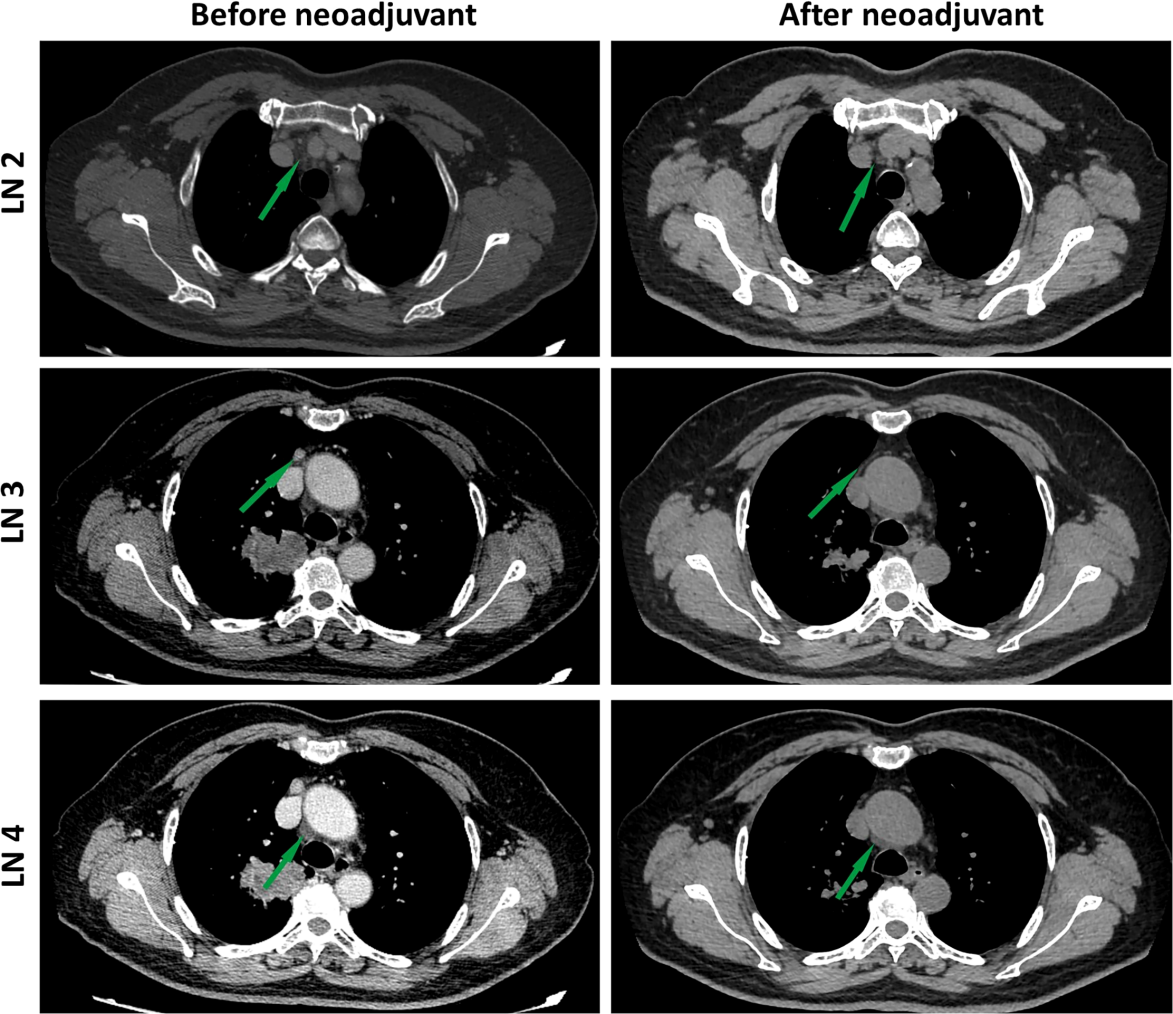
Images during neoadjuvant savolitinib treatment. Enhanced chest CT images of lymph nodes before and after the neoadjuvant therapy. The lymph nodes pointed by green arrows.

**Figure 3:**
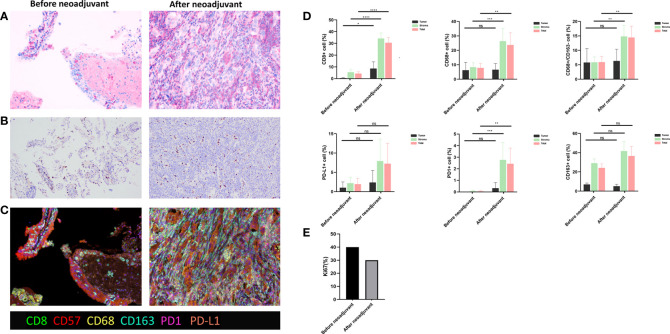
**(A)** Hematoxylin and eosin (HE) staining. **(B)** Ki67 staining. **(C)** Multiple immunohistochemistry staining, including CD8, CD57, CD68, CD163, PD-1, and PD-L1, before and after savolitinib neoadjuvant. **(D)** Quantitative analysis for staining data. ns, not significant. *p < 0.05; **p < 0.01; ***p < 0.001; ****p <0.0001; **(E)** Ki-67 index before and after savolitinib neoadjuvant.

After 4 weeks of treatment, a chest CT scan showed 60% tumor shrinkage and a partial decrease of mediastinal lymph nodes (**Figures 1**, **2**). The patient developed mild dizziness and nausea during savolitinib treatment. After an MDT discussion, right upper lobectomy and systemic lymphadenectomy were performed without any severe in-hospital complications. The final histopathological staging diagnosed pT2aN2M0 with occult lymph node metastasis in stations 2 (2/8). Lymph nodes at levels 3, 4, 7, 11, and 12 were negative for tumor involvement. The pathological examination showed that the Ki-67 index had dropped to 30% (**Figure 3**). There is extensive lymphocytic infiltration in tumors. Cholesterol crystals were observed as well as necrosis and fibrosis. The patient has been receiving savolitinib treatment post-surgery without radiation or chemotherapy. At the final follow-up in June 2022 (6 months after surgery), no grade 3/4 adverse events or disease progression had occurred. To determine any changes in the tumor immune environment due to savolitinib neoadjuvant treatment, we performed multiplexed immunohistochemistry (mIHC) on the biopsy tissue and the surgical specimen. The immunohistochemistry analysis indicated that the number of M1 macrophages, CD8^+^ tumor-infiltrating lymphocytes (TILs), and the level of PD-1 expression was increased significantly after neoadjuvant treatment, whereas PD-L1 amount remain unchanged. No CD57^+^ lymphocytes were detected before and after neoadjuvant therapy.

## Discussion

Neoadjuvant therapy is recommended to improve the survival rate of stage IIIA NSCLC patients. Previous reports have shown that neoadjuvant-targeted therapies are feasible for oncogene-positive NSCLC patients (6–8). We report the first case of neoadjuvant savolitinib treatment for NSCLC patients with METex14 skipping mutation.

In the present case, savolitinib exhibited a significant response in an NSCLC patient with METex14 skipping. The Ki-67 proliferation index decreased, and the tumor showed significant shrinkage after the savolitinib therapy, which was followed by successful lobectomy and systemic lymphadenectomy. Although radiological evaluation of most lymph nodes showed significant reduction after savolitinib neoadjuvant therapy, surgical pathology confirmed micrometastasis in lymph nodes [station 2 (2/8)]. Previous studies have reported that the response rate of CTONG 1103 (EMERGING) with a 42-day erlotinib neoadjuvant in EGFR-positive patients decreased, with a major pathologic response (MPR) of 9.7% and a lymph node downstaging rate of 10.8% (9). Thus, we assumed that savolitinib neoadjuvant treatment might achieve a longer median response than 4 weeks and better results if administered for a longer period before the operation.

Collectively in our case, neoadjuvant savolitinib may have converted the “cold” tumor to an immunologically “hot” tumor by recruiting CD8^+^ TILs and M1 macrophages. A previous study has reported that CD8^+^ TILs and NK cell populations decreased in patients with MET amplification, and it may be related to the MET signaling inducing the phosphorylation of UPF1 and downregulating the tumor cell STING expression (10). Thus, we proposed that the number of CD8^+^ TILs may increase after savolitinib treatment in METex14 NSCLC. Therefore, anti–PD-1 immunotherapy may be a viable treatment option for patients who have acquired resistance to savolitinib TKI treatment.

However, the efficacy of immunotherapy for METex14 NSCLC remains controversial. Some studies showed that immunotherapy might be effective for METex14 NSCLC patients. Mayenga et al. reported that 6 of 13 patients with METex14-mutated NSCLCs, who received immune checkpoint inhibitors (ICIs) treatment, had prolonged responses (11). Chen et al. reported that a METex14 NSCLC patient who developed targeted therapy resistance had a significant response to immunotherapy, which showed that immunotherapy might be a promising candidate for treating NSCLC patients with METex14 harboring MET-TKI–resistant mutations (12). In contrast, Sabari et al. reported that the overall response rate of METex14-altered lung cancers to PD-1/PD-L1 immune checkpoint inhibition was low and the median PFS was short. Neither PD-L1 status nor tumor mutation burden was correlated with response to immunotherapy (13). The ICIs efficacy in MET-mutated NSCLC was comparable to that observed in patients with pretreated unselected NSCLC in a retrospective, multicenter study (14). A phase II trial of capmatinib and spartalizumab versus capmatinib and placebo as first-line treatment for advanced NSCLC patients with METex14 skipping mutation is undergoing to evaluate the benefit of MET-TKI with ICIs in METex14 NSCLC (NCT04323436). Further prospective studies are necessary to define the role of ICIs in lung cancer patients with METex14 mutation.

In conclusion, our study showed that savolitinib neoadjuvant therapy is feasible for NSCLC patients with METex14 skipping mutation. Currently, an ongoing phase II trial of neoadjuvant and adjuvant capmatinib in NSCLC with METex14 skipping mutation is underway (NCT04926831). For NSCLC patients with METex14 skipping mutation, savolitinib as neoadjuvant treatment might provide a better option to replace chemotherapy. Further clinical trials are needed to evaluate the outcome and long-term prognosis of savolitinib in neoadjuvant therapy.

## Data availability statement

The original contributions presented in the study are included in the article/supplementary material. Further inquiries can be directed to the corresponding authors.

## Ethics statement

Written informed consent was obtained from the individual(s) for the publication of any potentially identifiable images or data included in this article.

## Author contributions

HZ: Data curation, Formal analysis, Writing Original draft preparation. YZ: Formal analysis, Resources Writing Original draft preparation. HQ: Data curation, Writing- Original draft preparation. JT: Resources. BZ: Visualization. NZ: Formal analysis. LL: Resources. ZQ: Conceptualization, Methodology, Supervision, Writing- Reviewing and Editing. CL: Resources, Conceptualization, Supervision, Validation. SX: Conceptualization, Validation, Visualization. Writing- Reviewing and Editing. All authors contributed to the article and approved the submitted version.

## Funding

The present study was funded by the National Natural Science Foundation of China (82172776), Tianjin Science and Technology Plan Project (19ZXDBSY00060), Tianjin Key Medical Discipline (Specialty) Construction Project, and Tianjin University training Program for Young and Middle‐Aged Backbone Innovative Talents (303078100412).

## Conflict of interest

The authors declare that the research was conducted in the absence of any commercial or financial relationships that could be construed as a potential conflict of interest.

## Publisher’s note

All claims expressed in this article are solely those of the authors and do not necessarily represent those of their affiliated organizations, or those of the publisher, the editors and the reviewers. Any product that may be evaluated in this article, or claim that may be made by its manufacturer, is not guaranteed or endorsed by the publisher.
